# Supplementary material for: Multi-generational koala pedigree analysis reveals rapid changes in heritable provirus load associated with life history traits
Source: Nat Commun. 2026 Jan 9;17:345. doi: 10.1038/s41467-025-66312-8 (PMC12789523; doi:10.1038/s41467-025-66312-8)
Supplement: Supplementary file 1 — Supplementary Information [file 41467_2025_66312_MOESM1_ESM.pdf]

Supplementary Materials

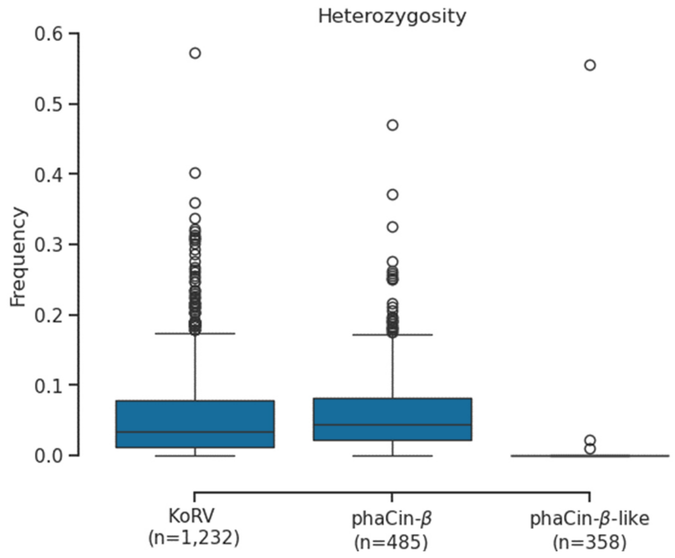

Supplementary Figure 1. Frequency of heterozygous integration sites in the San Diego Zoo Wildlife Alliance koala population for endogenous Koala Retrovirus (KoRV), phaCin-β, and phaCin-β-like. Numbers in parenthesis indicate the amount of integrations per endogenous retrovirus

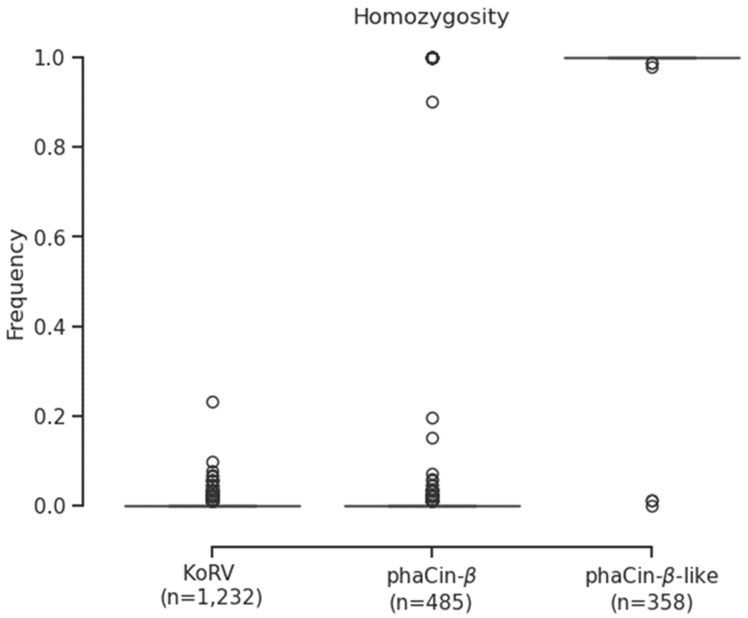

Supplementary Figure 2. Frequency of homozygous integration sites in the San Diego Zoo Wildlife Alliance koala population for endogenous Koala Retrovirus (KoRV), phaCin-β, and phaCin-β-like. Numbers in parenthesis indicate the amount of integrations per endogenous retrovirus

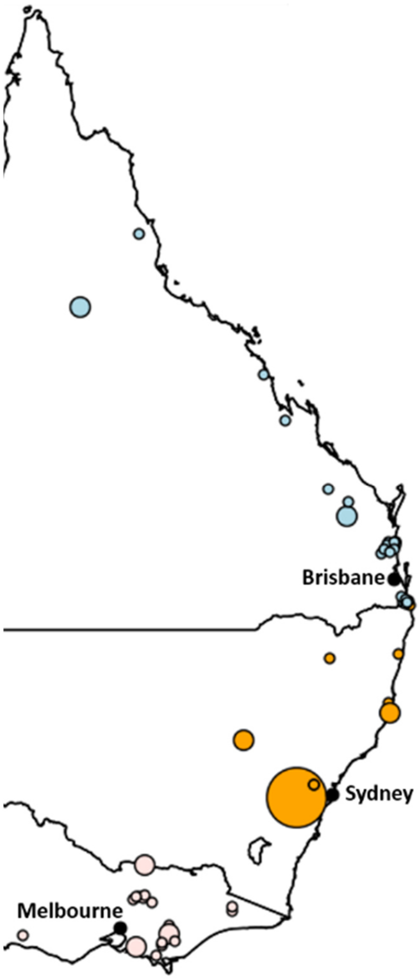

Supplementary Figure 3. Spatial distribution of wild koalas samples from the Koala Genome Survey database used in the current study across the eastern region of Australia. Circle sizes represent density of selected koalas in the different locations, while colors represent the states. Victoria is represented in pink, New South Wales in yellow, and Queensland in blue. Map created with Cartopy package in Python. Made with Natural Earth. Free vector and raster map data @ naturalearthdata.com.

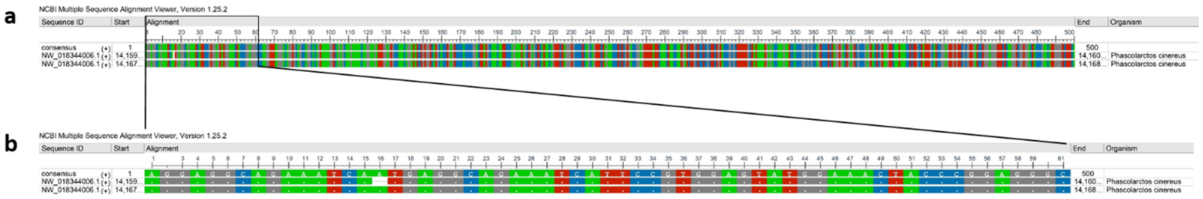

Supplementary Figure 4. Proviral LTR comparisons. (**a**) Alignment of 5'-LTR (NW_018344006.1 : 14,159,850 - 14,160,348) and 3'-LTR (NW_018344006.1 : 14,167,752 - 14,168,251) from the endogenous Koala Retrovirus located in the 9th intron of the *SLC29A1* (solute carrier family 29 member 1) gene. (**b**) One insertion is located at site 16 of 3'-LTR.

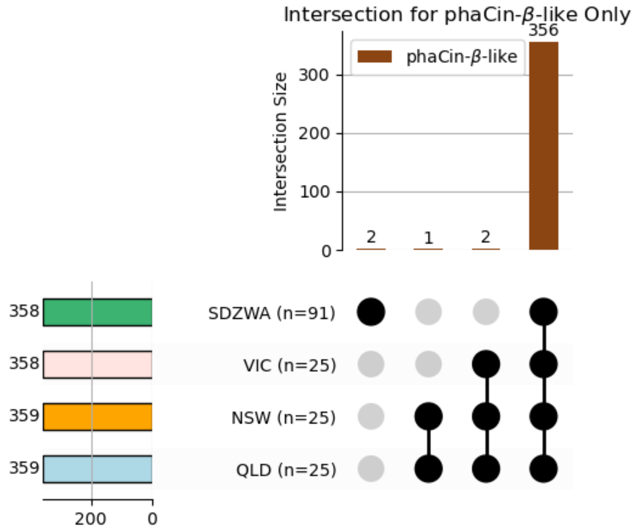

Supplementary Figure 5. Upset plot for the intersection between wild (populations from Queensland (QLD), New South Wales (NSW) and Victoria (VIC)), San Diego Zoo (SDZWA) and European Zoo (EUZ) populations for phaCin-β-like integrations. Numbers in parenthesis indicate number of animals per population used in the analysis. Circles filled in black represent the intersections, with respective bars indicating the number of phaCin-β-like shared between the marked populations. The left bar shows the total amount of phaCin-β-like observed for each population, while colors represent the different populations.

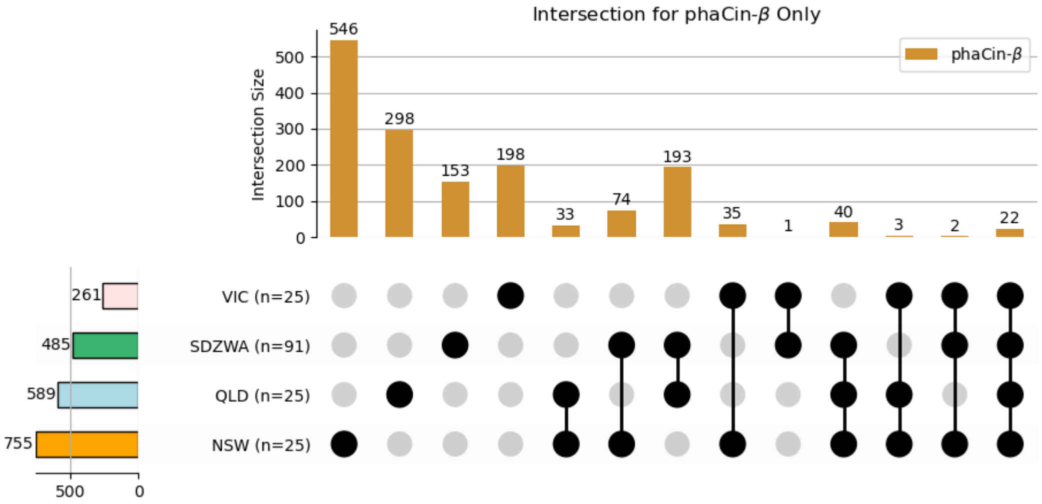

Supplementary Figure 6. Upset plot for the intersection between wild (populations from Queensland (QLD), New South Wales (NSW) and Victoria (VIC)), San Diego Zoo (SDZWA) and European Zoo (EUZ) populations for phaCin-β integrations. Numbers in parenthesis indicate number of animals per population used in the analysis. Circles filled in black represent the intersections, with respective bars indicating the number of phaCin-β shared between the marked populations. The left bar shows the total amount of phaCin-β observed for each population, while colors represent the different populations.

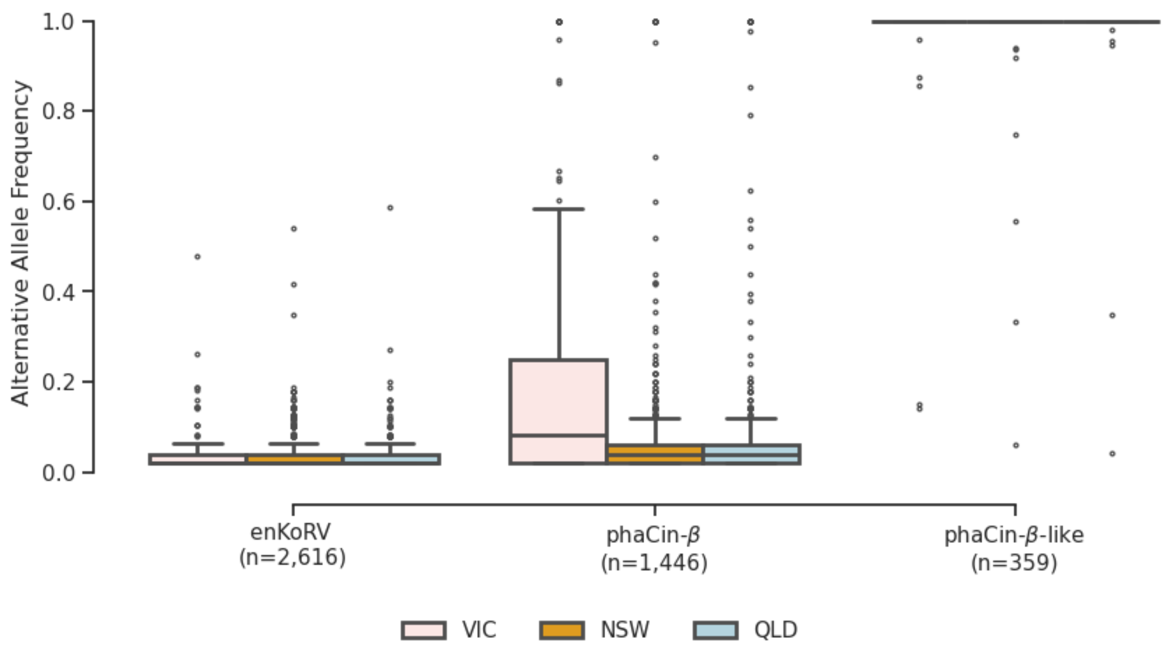

Supplementary Figure 7. Distribution of endogenous Koala Retrovirus (enKoRV), phaCin-β, and phaCin-β-like alternative allele frequencies for wild koalas in Victoria (VIC), New South Wales (NSW), and Queensland (QLD). The numbers in parenthesis indicate the amount of integrations per endogenous retrovirus type, while the colors represent the three populations.

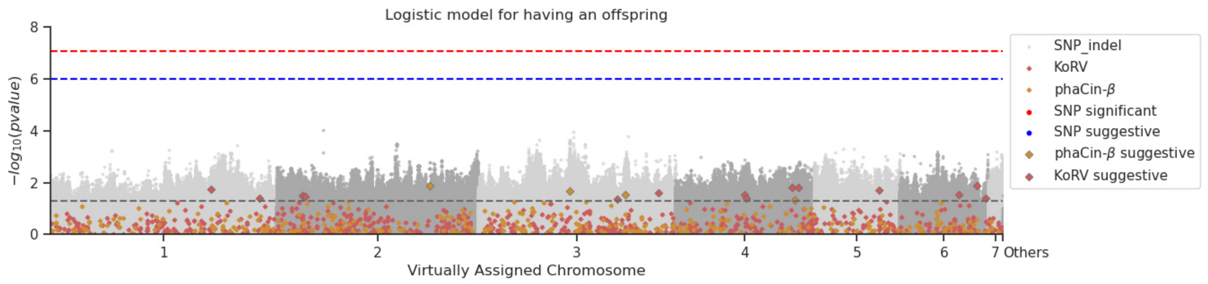

Supplementary Figure 8. Genome-wide association of endogenous retroviruses (ERV) and SNPs for a case-control model on reproduction success for koalas from the San Diego Zoo Wildlife Alliance. The negative logarithm base 10 of the p-values is plotted in the y-axis and the estimated genomic positions in the x-axis. The red line indicates a significant threshold and the blue a suggestive threshold for SNP association while the black line a suggestive threshold for ERVs. The color and format of the markers are described in the legend for suggestive and significant markers.

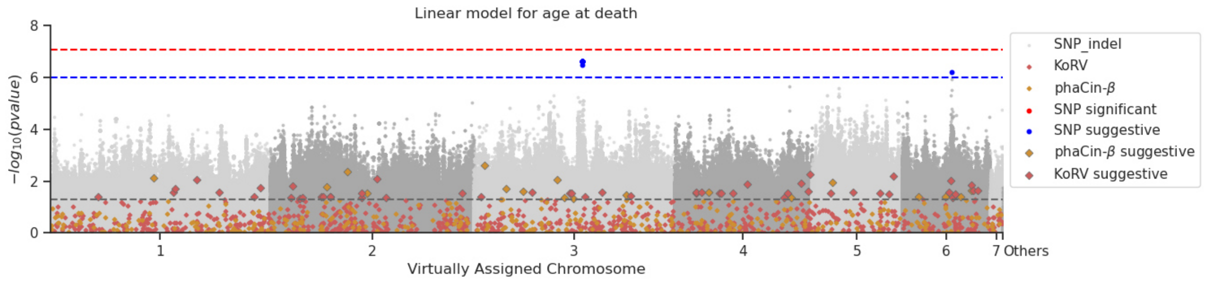

Supplementary Figure 9. Genome-wide association of endogenous retroviruses (ERV) and SNPs for a linear model on the age at death of koalas from the San Diego Zoo Wildlife Alliance. The negative logarithm base 10 of the p-values is plotted in the y-axis and the estimated genomic positions in the x-axis. The red line indicates a significant threshold and the blue a suggestive threshold for SNP association while the black line a suggestive threshold for ERVs. The color and format of the markers are described in the legend for suggestive and significant markers.

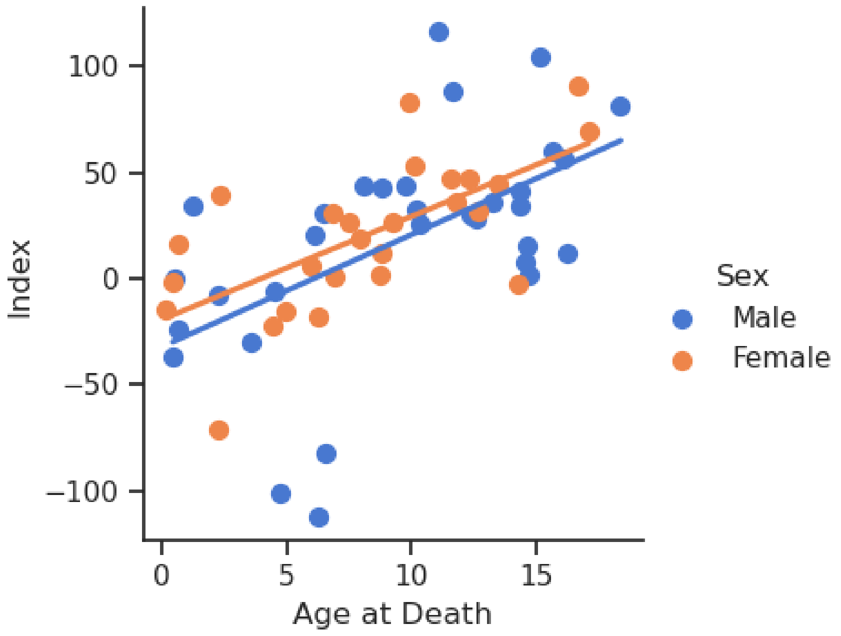

Figure 10. Distribution of age at death of koalas from San Diego Zoo Wildlife Alliance along breeding index estimated for longevity based on markers suggestively associated with age at death. Male koalas are colored in blue while female koalas in orange.

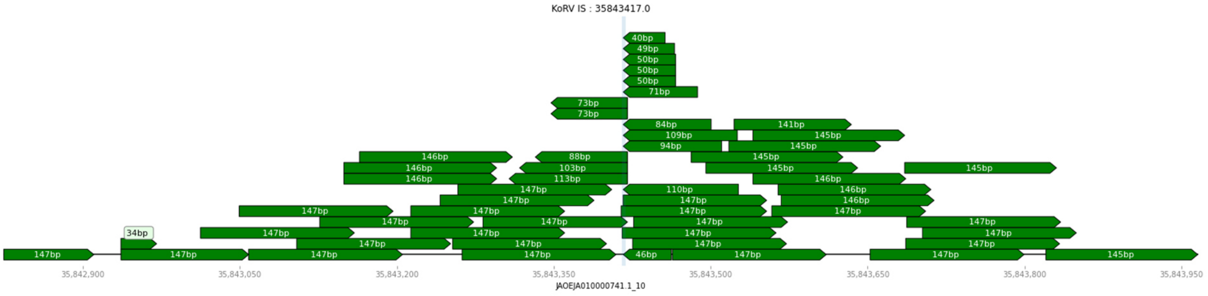

Supplementary Figure 11. Alignment of soft-clipped reads at the integration site (IS) 35,843,417 bp on contig JAOEJA010000741.1. All soft-clipped reads partially mapped to a KoRV strain and to the koala genome. Reads that were not soft-clipped had their mates aligned to a KoRV strain.

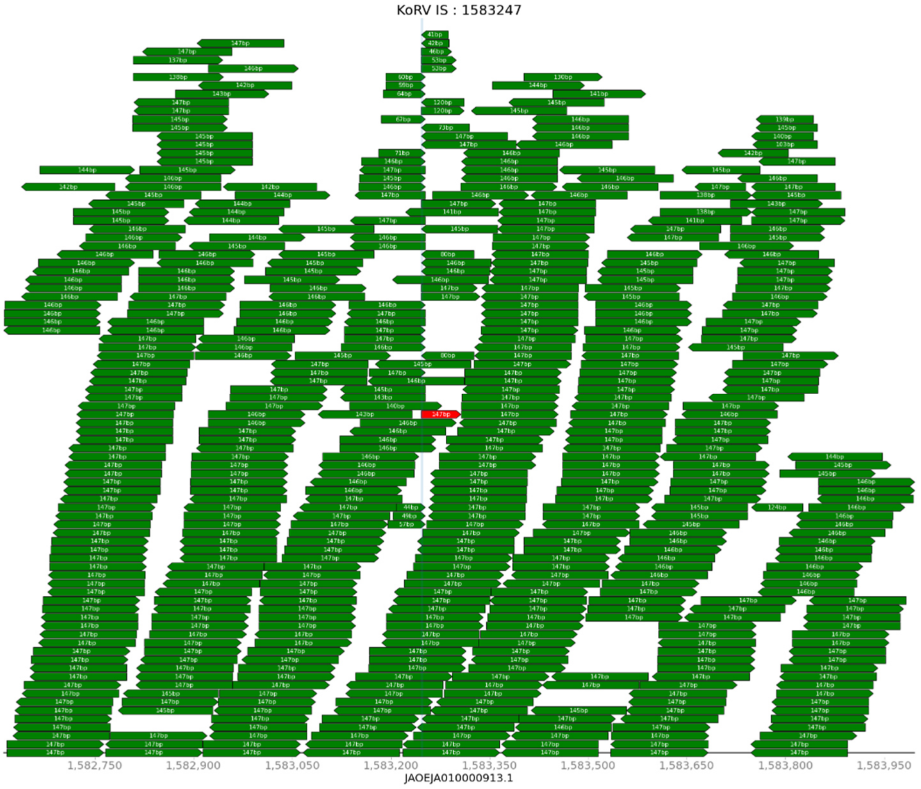

Supplementary Figure 12. Alignment of soft-clipped and non-soft-clipped reads at the insertion site (IS) 1,583,247 bp on contig JAOEJA010000913.1. The figure illustrates an example of a heterozygous enKoRV. Reads containing the viral sequence were soft-clipped around the ERV integration site, including the tandem duplication site (TDS), while reads without the viral sequence crossed the integration location.

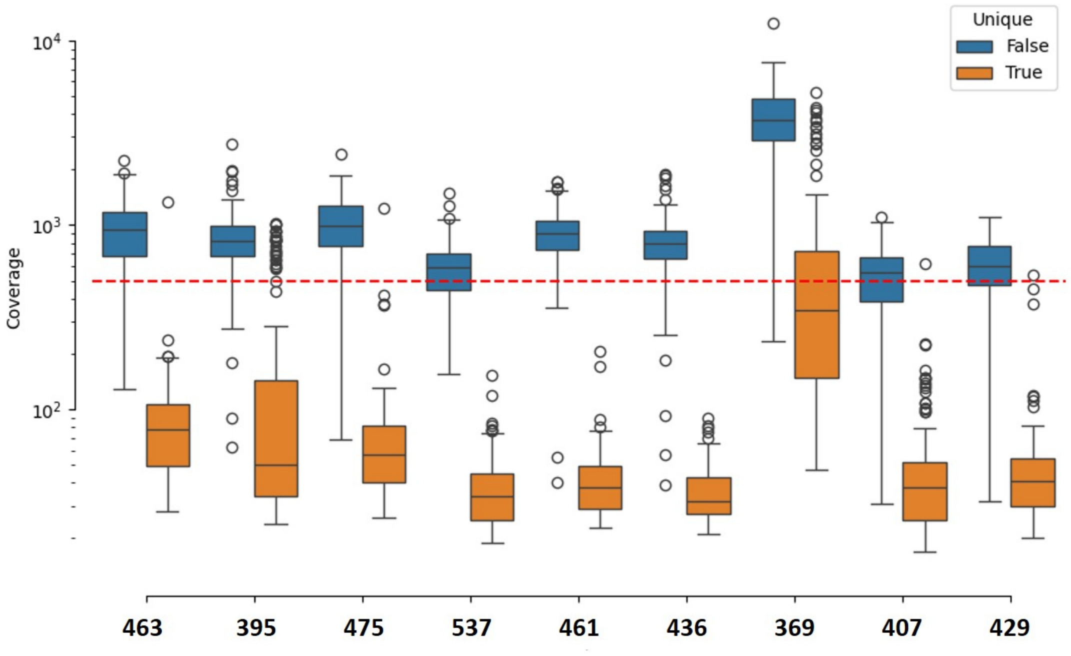

Supplementary Figure 13. Sequencing coverage of target sequenced koalas from European Zoos. Boxplots showing distribution of coverage (number of supporting reads for both breakpoints in log scale) in joeys, comparing Koala retroviruses also detected in parents (blue) and those that are unique to each joey (orange). An arbitrary cut-off of 500 reads (horizontal red line) was used to examine the highest coverage of retroviruses unique to joeys, except for 369, where a cut-off of 1,000 reads was used.

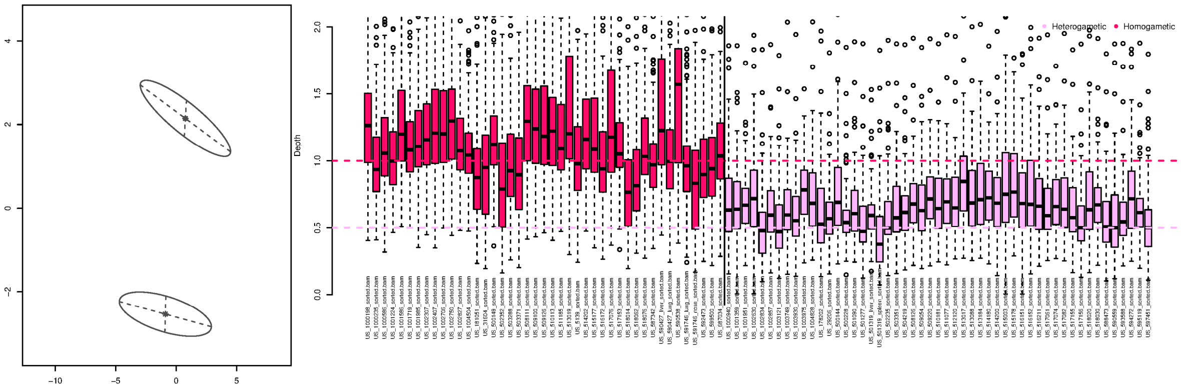

Supplementary Figure 14. Detection of sex scaffolds in koalas. Gaussian mixture clustering of koalas by sex using the SATC software. On the left, a PCA plot of normalized depth across all scaffolds with the inferred Gaussian mixture components shown as ellipsoids scales by its variance is presented. On the right, a boxplot of normalized depth of sex-linked scaffolds for each sample is shown. The samples are ordered based on the inferred two sex group. Expected values for each group are shown by horizontal dashed lines of 0.5 (heterogametic) and 1.0 (homogametic). A vertical line is to separate between heterogametic and homogametic samples.

Supplementary Note 1: Virtual Assignment of Chromosomes

Scaffolds were virtually assigned to chromosomes based on the chromosomes annotation of the common wombat genome (GCA_028626985.1). Each scaffold was blasted (blastn v2.12.0) against the wombat autosomes, being assigned to the chromosome with longest scaffold alignment, covering at least 90% of the koala scaffold length. The sex chromosomes were defined separately (Supplementary Data 8 and Supplementary Figure 14), using the SATC v1 software [1]. The order of the scaffolds follow the order in which they were aligned in the wombat genome, and is used for visual representation only. The genomic positions, however, are still reported per scaffold.

Supplementary Note 2: enKoRV diversity

In addition to host SNPs and ERVs, enKoRV SNPs were also detected for wild koalas and koalas from SDZWA. One hundred and eleven high-quality biallelic SNPs were found (Supplementary Data 9). Most SNPs were found at low frequency, with an average AAF of 0.05 (SD 0.12). A series of SNPs were specific to one population, or much more frequent in one population than in the others. This was particularly observed for the intergenic region (gag leader sequence) and for the *gag-pol* gene of the KoRV-A genome (Supplementary Figure 15a). As expected, due to the genetic bottleneck in southern Australia, VIC koalas demonstrated some enKoRV SNPs with much higher frequency than observed elsewhere. With the exception of SDZWA and QLD, the populations could be differentiated based on the enKoRV SNPs (Supplementary Figure 15b).

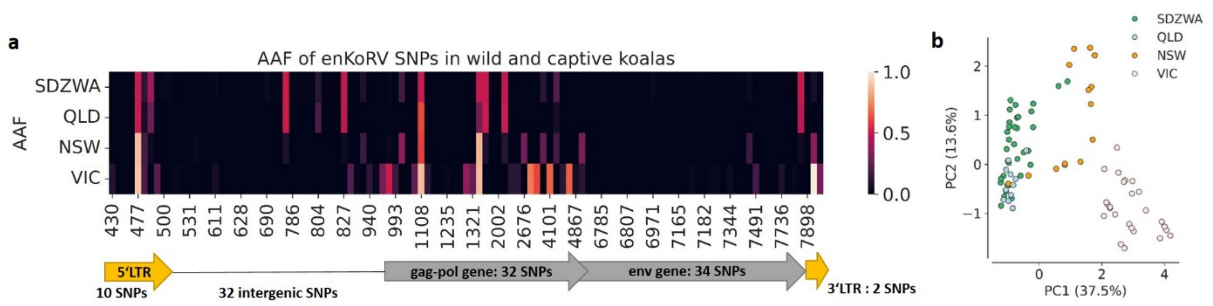

Supplementary Figure 15. SNPs detected for enKoRV in captive and wild koalas. (**a**) Heatmap for the alternative allele frequency (AAF) of SNPs detected across the KoRV-A genome. KoRV-A positions are shown in the x-axis, together with a schematic annotation of the KoRV-A genome. Length of genomic features not to scale. (**b**) Principal component analysis using enKoRV SNPs.

Although the number of SNPs was very similar between the gag leader sequence, *gag-pol* gene, and *env* gene, considering the length of those regions (LTRs are 505 bp long each, *gag-pol* is 5,064 bp long, and *env* gene is 1,980 bp long), the substitutions per nucleotide rates differ considerably. The rate is 0.020 for the 5'LTR region, 0.069 for the gag leader sequence, 0.006 for the *gag-pol* gene, 0.017 for the *env* gene, and 0.004 for the 3'LTR.

Moreover, when examining association between enKoRV SNPs and neoplasia, no significant or suggestive association was observed.

1. Nursyifa, C., Brüniche-Olsen, A., Garcia-Erill, G., Heller, R. & Albrechtsen, A. Joint identification of sex and sex-linked scaffolds in non-model organisms using low depth sequencing data. *Mol Ecol Resour* **22**, 458–467 (2022).
2. Cartopy. v0.24.1. 09-Oct-2024. Met Office. UK. https://cartopy.readthedocs.io/stable/
