# Supplementary material for: Multi-generational koala pedigree analysis reveals rapid changes in heritable provirus load associated with life history traits
Source: Nat Commun. 2026 Jan 9;17:345. doi: 10.1038/s41467-025-66312-8 (PMC12789523; doi:10.1038/s41467-025-66312-8)
Supplement: Supplementary file 2 — Description of Additional Supplementary Files [file 41467_2025_66312_MOESM2_ESM.pdf]

Title: Supplementary Data 1
Description: Sample information for deceased and alive koalas from San Diego Zoo Wildlife Alliance.

Title: Supplementary Data 2
Description: Sample information for koalas from European Zoos.

Title: Supplementary Data 3
Description: Number of wild koalas analyzed per location. Subset of the Koala Genome Survey Database.

Title: Supplementary Data 4
Description: ERVs that did not persist in the current captive population (AAF-deceased ≥ 0.05).

Title: Supplementary Data 5.
Description: ERVs that exhibited increased frequency of death by cancer in 10%.

Title: Supplementary Data 6
Description: Genome-wide association results. Tested phenotypes include: age at death (AgeDead), cause of death by any type of cancer (Cancer), cause of death by any type of cancer except leukemia (CancerWOLeukemia), cause of death by any type of cancer except leukemia whereas controls include only non-cancer related deaths (CancerWOLeukemiaStrictControl), cause of death by any leukemia (Leukemia), cause of death by leukemia whereas controls include only non-cancer related deaths (LeukemiaStrictControl), reproducion success including alive and deceased koalas (OffspringAliveBinDead), and reproducion success including only deceased koalas (OffspringTotalBinDead). Allel frequencies (AF) for the minor alleles are provided. For linear models, the β‑coefficient (effect per allele) is provided, and for logistic models, the log(odds ratio). P-values (P) were obtained from two-sided Wald test and are shown without multiple testing correction.

Title: Supplementary Data 7
Description: Annotation of enKoRV-A SNPs. RAF= reference allele frequency, AAF= alternative allele frequency.

Title: Supplementary Data 8
Description: Contigs linked to sex chromosomes assigned by SATC v1. P-values were obtained from two-sided Welch's two-sample t-tests, comparing mean values between groups. P-values were not corrected for multiple testing.

Title: Supplementary Data 9
Description: Association results for enKoRV SNPs. For reproduction success, the provided estimate is log(odds ratio), and for age at death the β-coefficient (effect per

allele). P-values (P) were obtained from two-sided Wald test and are shown without multiple testing correction.
