# Supplementary material for: Multi-generational koala pedigree analysis reveals rapid changes in heritable provirus load associated with life history traits
Source: Nat Commun. 2026 Jan 9;17:345. doi: 10.1038/s41467-025-66312-8 (PMC12789523; doi:10.1038/s41467-025-66312-8)
Supplement: Supplementary file 4 — Reporting Summary [file 41467_2025_66312_MOESM4_ESM.pdf]

nature portfolio

Corresponding author(s): Alex Greenwood

Last updated by author(s): Oct 10, 2025

Reporting Summary

Nature Portfolio wishes to improve the reproducibility of the work that we publish. This form provides structure for consistency and transparency in reporting. For further information on Nature Portfolio policies, see our Editorial Policies and the Editorial Policy Checklist.

Statistics

For all statistical analyses, confirm that the following items are present in the figure legend, table legend, main text, or Methods section.

n/a	Confirmed	
☐	☒	The exact sample size (n) for each experimental group/condition, given as a discrete number and unit of measurement
☐	☒	A statement on whether measurements were taken from distinct samples or whether the same sample was measured repeatedly
☐	☒	The statistical test(s) used AND whether they are one- or two-sided<br>*Only common tests should be described solely by name; describe more complex techniques in the Methods section.*
☐	☒	A description of all covariates tested
☐	☒	A description of any assumptions or corrections, such as tests of normality and adjustment for multiple comparisons
☐	☒	A full description of the statistical parameters including central tendency (e.g. means) or other basic estimates (e.g. regression coefficient) AND variation (e.g. standard deviation) or associated estimates of uncertainty (e.g. confidence intervals)
☐	☒	For null hypothesis testing, the test statistic (e.g. F, t, r) with confidence intervals, effect sizes, degrees of freedom and P value noted<br>*Give P values as exact values whenever suitable.*
☒	☐	For Bayesian analysis, information on the choice of priors and Markov chain Monte Carlo settings
☒	☐	For hierarchical and complex designs, identification of the appropriate level for tests and full reporting of outcomes
☐	☒	Estimates of effect sizes (e.g. Cohen's d, Pearson's r), indicating how they were calculated

Our web collection on statistics for biologists contains articles on many of the points above.

Software and code

Policy information about availability of computer code

Data collection	*Provide a description of all commercial, open source and custom code used to collect the data in this study, specifying the version used OR state that no software was used.*
Data analysis	fastp v0.22.0, BWA-mem v2-2.2.1, samtools v1.7, RepeatMasker v4.1.2-p1, dragmap v1.3.0, GATK v4.4.0, BLAST v2.12.0, SMRT®Portal, Minimap v2.1, fastqc v0.12.1, hisat2 v2.2.1, htseq-count v2.0.8, DESeq2 v4.1.3, SciPy v1.11.4 , Scikit-learn v.1.5, R v4.1.1, Python v.3.12, cartopy v0.24, Geneious Prime v2025.0.2, PLINK v2, Ensembl Variant Effect Predictor release 105 , Miro for pedigree visualization (https://miro.com/). All scripts available at https://github.com/neumannguib/Koala_SDZWA.

For manuscripts utilizing custom algorithms or software that are central to the research but not yet described in published literature, software must be made available to editors and reviewers. We strongly encourage code deposition in a community repository (e.g. GitHub). See the Nature Portfolio guidelines for submitting code & software for further information.

Data

Policy information about availability of data

All manuscripts must include a data availability statement. This statement should provide the following information, where applicable:

- Accession codes, unique identifiers, or web links for publicly available datasets
- A description of any restrictions on data availability
- For clinical datasets or third party data, please ensure that the statement adheres to our policy

Publicly available data from the Koala Genome Survey (https://awgg-lab.github.io/australasiangenomes/species/Phascolarctos_cinereus.html) and RNA-Seq data

from five KoRV-free koala individuals was used (European Nucleotide Archive, project PRJEB21505). Data generated in the current work are deposited at European Nucleotide Archive, under the project PRJEB86269.

Research involving human participants, their data, or biological material

Policy information about studies with human participants or human data. See also policy information about sex, gender (identity/presentation), and sexual orientation and race, ethnicity and racism.

Reporting on sex and gender	*Use the terms sex (biological attribute) and gender (shaped by social and cultural circumstances) carefully in order to avoid confusing both terms. Indicate if findings apply to only one sex or gender; describe whether sex and gender were considered in study design; whether sex and/or gender was determined based on self-reporting or assigned and methods used.*<br>*Provide in the source data disaggregated sex and gender data, where this information has been collected, and if consent has been obtained for sharing of individual-level data; provide overall numbers in this Reporting Summary. Please state if this information has not been collected.*<br>*Report sex- and gender-based analyses where performed, justify reasons for lack of sex- and gender-based analysis.*
Reporting on race, ethnicity, or other socially relevant groupings	*Please specify the socially constructed or socially relevant categorization variable(s) used in your manuscript and explain why they were used. Please note that such variables should not be used as proxies for other socially constructed/relevant variables (for example, race or ethnicity should not be used as a proxy for socioeconomic status).*<br>*Provide clear definitions of the relevant terms used, how they were provided (by the participants/respondents, the researchers, or third parties), and the method(s) used to classify people into the different categories (e.g. self-report, census or administrative data, social media data, etc.)*<br>*Please provide details about how you controlled for confounding variables in your analyses.*
Population characteristics	*Describe the covariate-relevant population characteristics of the human research participants (e.g. age, genotypic information, past and current diagnosis and treatment categories). If you filled out the behavioural & social sciences study design questions and have nothing to add here, write "See above."*
Recruitment	*Describe how participants were recruited. Outline any potential self-selection bias or other biases that may be present and how these are likely to impact results.*
Ethics oversight	*Identify the organization(s) that approved the study protocol.*

Note that full information on the approval of the study protocol must also be provided in the manuscript.

Field-specific reporting

Please select the one below that is the best fit for your research. If you are not sure, read the appropriate sections before making your selection.

☐ Life sciences ☐ Behavioural & social sciences ☒ Ecological, evolutionary & environmental sciences

For a reference copy of the document with all sections, see nature.com/documents/nr-reporting-summary-flat.pdf

Ecological, evolutionary & environmental sciences study design

All studies must disclose on these points even when the disclosure is negative.

Study description	This study uses an extensive koala pedigree with associated individual koala metadata to achieve insights into the retroviral colonization process of vertebrate genomes and associated health impacts.<br>We analysed sequencing data of 55 koala triads, providing a novel and unique dataset for the identification of new genomic structural variation involving endogenous retroviruses. We examined three of the youngest germline colonizing viruses known including the Koala Retrovirus (KoRV), phaCin-ß, and phaCin-ß-like observed within captive and wild koalas. We were able to establish a koala specific mutation rate to more accurately estimate the colonization times of the retroviruses. We were able to observe the generation and elimination of endogenous retroviruses and associated these processes with impacts to koala health, providing valuable information that could influence conservation efforts and genetic studies of endangered species.
Research sample	186 koala individuals were included in this study: 91 koalas from San Diego Zoo Wildlife Alliance, 20 koalas from European zoos, and 75 wild koalas from the Koala Genome Survey
Sampling strategy	All available koala samples from the zoos were used. For wild koalas, 25 samples per region were used, considering a sample size comparable to 20 samples for European zoos, and a recommended sample size of 25 for genetic diversity analyses.
Data collection	Blood or tissue were collect from veterinarians employed at the different zoos
Timing and spatial scale	Available samples were collected between 1992 to 2023 and preserved frozen at -80 °C until DNA extraction
Data exclusions	No data were excluded from the analyses

Reproducibility	Three duplicates were available and used to validate the detection and zygosity characterization of ERVs. In addition, mendelian error was checked for the triads in order to verify the detection reliability per ERV. Most analyses follow standard sequencing procedures, using open-source data. Customized code is available on GitHub.
Randomization	All available koala samples from the zoos were used. For specific analyses such as for the genome-wide association study, population structure and sire were verified as cofactors. For the comparison with wild koalas, samples were taken from all different available regions and subregions.
Blinding	Blinding was not necessary since our study applied quantitative and objective measurements, and no extensive knowledge about individual animals was available

Did the study involve field work? ☐ Yes ☒ No

Reporting for specific materials, systems and methods

We require information from authors about some types of materials, experimental systems and methods used in many studies. Here, indicate whether each material, system or method listed is relevant to your study. If you are not sure if a list item applies to your research, read the appropriate section before selecting a response.

Materials & experimental systems

n/a	Involved in the study
☒	☐ Antibodies
☒	☐ Eukaryotic cell lines
☒	☐ Palaeontology and archaeology
☐	☒ Animals and other organisms
☒	☐ Clinical data
☒	☐ Dual use research of concern
☒	☐ Plants

Methods

n/a	Involved in the study
☒	☐ ChIP-seq
☒	☐ Flow cytometry
☒	☐ MRI-based neuroimaging

Animals and other research organisms

Policy information about studies involving animals; ARRIVE guidelines recommended for reporting animal research, and Sex and Gender in Research

Laboratory animals	NA
Wild animals	Captive koalas from European zoos and San Diego Zoo - Wild animals data retrived from publicly available database
Reporting on sex	Sex was available and tested as cofactor in the corresponding genome-wide association study section
Field-collected samples	NA
Ethics oversight	Blood from living koalas from the San Diego Zoo Wildlife Alliance (SDZWA) were sampled in accordance with the Institutional Animal Care and Use Committee (IACUC) under IACUC proposals 15-017, 18-024 and 21-019 "Opportunistic Sample Collection during Veterinary Procedures". Samples from deceased animals were taken opportunistically upon death of the animal and stored in the frozen tissues collection of the SDZWA. Blood was taken from live animals in the European collections during regular veterinary care, according to each national regulations and institution code of ethic, or opportunistically upon the death of an individual koala. The collections were conducted upon request of the coordinator (Kerstin Ternes) and the veterinary advisor (Baptiste Mulot) of the European Association of Zoos and Aquaria (EAZA) Ex Situ Programme (EEP), in compliance with EAZA ethical standards."<br><br>Wild koala sample data was obtained from the open access Koala Genome Survey and scientific and ethics documentation from that project are listed within the following publication Hogg, C. J., Silver, L., McLennan, E. A., & Belov, K. (2023). Koala genome survey: an open data resource to improve conservation planning. Genes, 14(3), 546.

Note that full information on the approval of the study protocol must also be provided in the manuscript.

Plants

Seed stocks	*Report on the source of all seed stocks or other plant material used. If applicable, state the seed stock centre and catalogue number. If plant specimens were collected from the field, describe the collection location, date and sampling procedures.*
Novel plant genotypes	*Describe the methods by which all novel plant genotypes were produced. This includes those generated by transgenic approaches, gene editing, chemical/radiation-based mutagenesis and hybridization. For transgenic lines, describe the transformation method, the number of independent lines analyzed and the generation upon which experiments were performed. For gene-edited lines, describe the editor used, the endogenous sequence targeted for editing, the targeting guide RNA sequence (if applicable) and how the editor was applied.*
Authentication	*Describe any authentication procedures for each seed stock used or novel genotype generated. Describe any experiments used to assess the effect of a mutation and, where applicable, how potential secondary effects (e.g. second site T-DNA insertions, mosiacism, off-target gene editing) were examined.*
